# Supplementary material for: The B22 Dilemma: Structural Basis for Conformational Differences in Proinsulin B-Chain Arg22 Mutants
Source: Biomolecules. 2025 Apr 12;15(4):577. doi: 10.3390/biom15040577 (PMC12025217; doi:10.3390/biom15040577)
Supplement: Supplementary file 1 [file biomolecules-15-00577-s001.zip › biomolecules-3529952-supplementary.pdf]

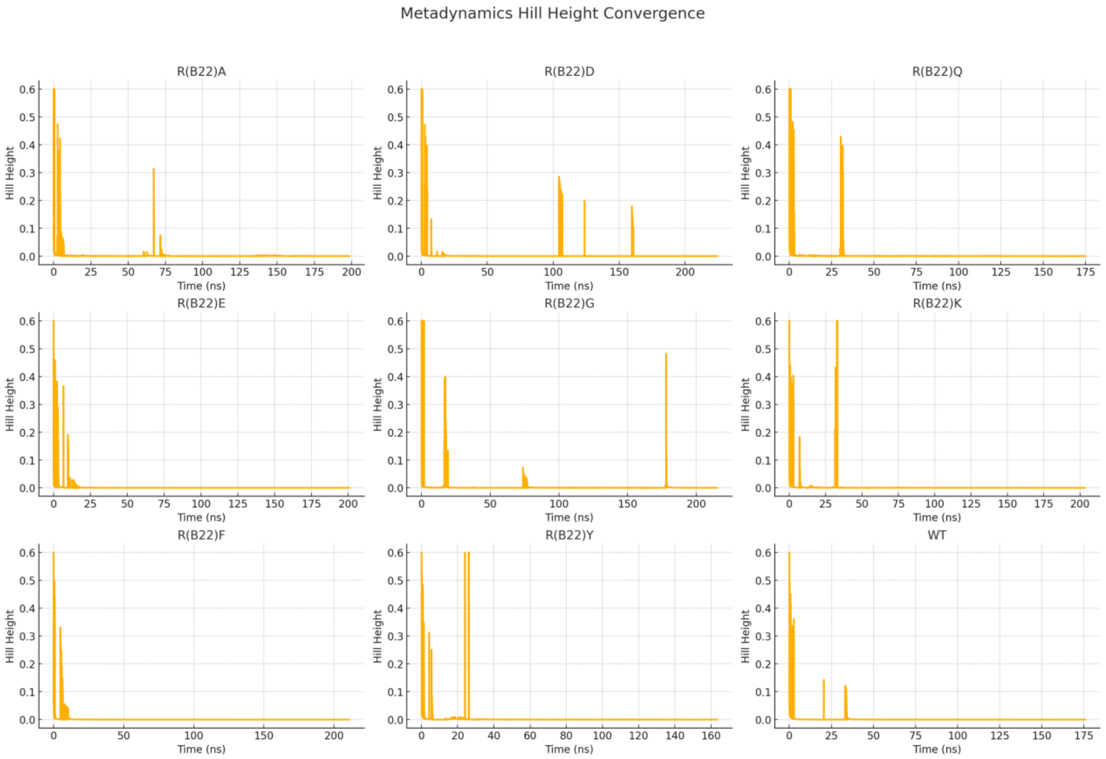

Figure S1: Time evolution of metadynamics hill heights for wild-type (WT) and Arg22 mutants during well-tempered metadynamics simulations. Each panel shows the hill height (in kcal/mol) as a function of simulation time (in nanoseconds) for a specific variant, where Arg22 (R) in the B-chain is mutated to another residue (denoted as R(B22)X). The decay and stabilization of hill heights over time indicate the convergence behavior of the biasing potential in each system.
